# Supplementary material for: Glycoproteins Presenting Galactose and N-Acetylgalactosamine in Human Seminal Plasma as Potential Players Involved in Immune Modulation in the Fertilization Process
Source: Int J Mol Sci. 2021 Jul 8;22(14):7331. doi: 10.3390/ijms22147331 (PMC8303229; doi:10.3390/ijms22147331)
Supplement: Supplementary file 1 [file ijms-22-07331-s001.zip › ijms-1285200-supplementary/Supplemental Table S1.pdf]

Table S1. LC-MS protein identification of lectin-reactive bands separated by SDS-PAGE

Fraction	Protein	Score	Matches	Molar Mass [Da]
135 kDa	Mucin 6	12688	271	263,159
	Aminopeptidase N	8732	204	109,870
	Serum albumin	5456	111	71,317
	Fibronectin	5109	111	266,052
	Lactotransferrin	4699	96	80,014
	Dipeptidyl peptidase 4	3143	75	88,907
	Laminin subunit beta 2	2410	56	202,982
	Beta mannosidase	1174	31	101,800
	Cation independent mannose 6 phosphate receptor	984	25	281,155
	Neogenin	864	13	160,658
	Nicastrin	725	13	79,103
83 kDa	Lactotranferrin	43323	872	83,000
	Fibronectin	14382	309	266,052
	Serum albumin	4267	108	71,317
	Serotransferrin	2896	78	79,294
	Mucin-6	2692	62	263,159
	Polymeric immunoglobulin receptor	1972	37	84,429
	Alpha 2 macroglubulin	1049	32	164,613
	Calsyntenin-1	529	13	110,978
	Leukemia inhibitory factor receptor	465	17	125,090
	Neprilysin	451	17	86,144
	Aminopeptydaza N	385	8	109,870
	Alpha N acetyloglucosaminidase	346	6	82,670
	Matrix remodelling associated protein 5	338	7	314,065
	Interleukin 6 receptor subunit beta	333	12	104,498
	Heat shock protein HSP 90 beta	310	9	83,554
	Matrilin 2	264	8	110,644
	Heat shock protein HSP 90 alpha	196	7	85,006
	Collectin-12	186	7	82,035
70 kDa	Serum albumin	38789	946	71,317
	Galectin 3 binding protein	2960	59	66,202
	Lactotransferrin	1668	44	80,014
	Fibronectin	1061	41	266,052
	Carboxipeptidase Z	640	20	74,577
	Mucin-6	482	17	263,159
	Cartilage acidic protein 1	299	10	72,174
	Plastin 2	281	8	70,814
	Growth arrest specific protein 6	254	11	81,678
	Hornerin	246	7	283,140
	Heat shock 70 kDa protein 1A	238	6	70,294
	Transketolase	176	4	68,519
53 kDa	Prostatic acid phosphatase	12624	443	44,880
	Alpha 1 antitrypsin	3616	139	46,878
	Fibronectin	1528	46	266,052
	Lactotransferrin	1390	42	80,014
	Hornerin	814	21	283,140
	Gluthatione synthetase	687	25	52,523
	IgGFc binding protein	574	19	596,443

	Extracellular matrix protein 1	493	16	62,232
	Mucin 6	485	18	263,159
	Alpha 2 antiplasmin	434	15	54,873
	Vitamin D binding protein	349	8	54,526
	Clusterin	335	8	53,031
	Hemopexin	303	5	52,385
	Gluthatione reductase	240	5	56,791
	Beta hexosaminidase subunit beta	230	10	63,527
	Angiotensinogen	228	7	53,406
	Solute carrier family 2, glucose transporter member	184	6	55,394
	Galectin 3 binding protein	184	7	66,202
	Desmoplakin	172	6	334,021
	Aspartyl aminopeptidase	156	3	53,022
32 kDa	Fibronectin	9291	221	266,052
	Prostate specific antigen	6171	222	29,293
	Annexin	4182	80	35,971
	Clusterin	2358	66	53,031
	Prostatic acid phosphatase	1381	35	44,880
	Prosaposin	1263	42	59,899
	Serum albumin	1098	42	71,317
	Carboxypeptidase E	1059	34	53,516
	Cysteine rich secretory protein 1	1026	19	29,432
	Purine nucleoside phosphorylase	1010	23	32,325
	Lactotransferrin	891	26	80,014
	Protein glutamine gamma glutamyltransferase 4	767	19	77,951
	Zinc alpha 2 glycoprotein	706	17	34,465
	Creatine kinase B type	652	12	42,902
	Hornerin	651	20	283,140
	Syntenine 1	484	14	32,595
	Plasma serine protease inhibitor	400	16	45,760
	Extracellular matrix protein 1	390	10	62,232
	Heat shock protein HSP 90 alpha	373	10	85,006
	Sulfhydryl oxidase 1	351	13	83,324
	L lactate dehydrogenase B	321	5	36,900
	SPARC like protein 1	316	8	76,017
	Complement factor B	300	14	86,847
	Immunoglobulin gamma 1 heavy chain	280	15	49,925
	Galectin 3 binding protein	253	5	66,202
	Mucin 6	249	12	263,159
	Complement C4	204	12	194,261
	Glycodelin	176	4	20,953
	Epididymal sperm binding protein 1	129	5	27,001
	Testis expressed protein 101	77	1	27,504
15 kDa	Prolactin inducible protein	37959	916	16,847
	Peptidyl prolyl cis trans isomerase A	1145	33	18,229
	Prostatic acid phosphatase	1084	37	44,880
	Prosaposin	992	31	59,899
	Serum albumin	937	25	71,317
	Prostate specific antigen	910	31	29,293
	Cathelicidin antimicrobial peptide	901	32	19,517

	Fibronectin	814	30	266,052
	Ras related protein Rab 3B	727	17	24,970
	Nucleoside diphosphate kinase B	663	21	17,401
	Carbonic anhydrase 4	615	19	35,295
	Caspase 14	472	11	27,947
	Clusterin	441	11	53,031
	Nucleoside diphosphate kinase 3	431	12	19,231
	Elongation factor 1 gamma	426	10	50,429
	Peroxiredoxin-5, mitochondrial	422	9	22,301
	Cystatin M	279	8	16,785
	Lipocalin 1	268	10	19,409
	Cofilin 1	263	5	18,719
	Semenogelin 1	241	8	52,157
	Cystatin C	239	9	16,017
	CD59 glycoprotein	238	9	14,795
	Transthyretin	228	4	15,991
	Semenogelin 2	143	6	65,519
<15 kDa	Prolactin inducible protein precursor	1823	39	16,847
	Semenogelin 1 preproprotein	1134	27	52,157
	Prolactin induced protein	1090	23	9,232
	Semenogelin 2 precursor	1023	21	65,519
	Fibronectin isoform 3 preproprotein	418	8	262,656
	Prostate specific antigen precursor	286	7	28,780
