# Supplementary material for: Glycoproteins Presenting Galactose and N-Acetylgalactosamine in Human Seminal Plasma as Potential Players Involved in Immune Modulation in the Fertilization Process
Source: Int J Mol Sci. 2021 Jul 8;22(14):7331. doi: 10.3390/ijms22147331 (PMC8303229; doi:10.3390/ijms22147331)
Supplement: Supplementary file 1 [file ijms-22-07331-s001.zip › ijms-1285200-supplementary/Supplemental Table S2.pdf]

Table S2. LC-MS identification of proteins isolated in lectin affinity chromatography.

VVL			
Protein identified	Score	Matches	Molar mass (Da)
Semenogelin-2	2997	135	65,497
Prolactin-inducible protein	1482	39	16,792
Semenogelin-1	1278	49	52,146
Lactotransferrin	50	16	79,650
Cystatin-C	108	1	15,973
Prostate-specific antigen	86	2	29,183
Fibronectin	67	2	275,047
Immunoglobulin heavy constant alpha 1	64	1	38,320
Thioredoxin	60	1	11,960
Filaggrin-2	56	1	249,032
Prostatic acid phosphatase	55	1	44,813
Beta-microseminoprotein	49	1	13,362
Metalloproteinase inhibitor 1	43	1	23,707
Aminopeptidase N	43	1	109,793
Antileukoproteinase	43	1	15,052
Zinc-alpha-2-glycoprotein	40	1	34,421

MPL			
Protein identified	Score	Matches	Molar mass (Da)
Prolactin-inducible protein	9346	158	16,792
Semenogelin-2	6473	159	65,497
Semenogelin-1	3895	80	52,146
Fibronectin	1471	28	275,047
Lactotransferrin	1440	29	79,650
Albumin	897	18	70,931
Prostate-specific antigen	809	21	29,183
Aminopeptidase N	758	16	109,793
Prostatic acid phosphatase	539	11	44,813
60 kDa heat shock protein, mitochondrial	467	5	61,154
Cystatin-C	412	5	15,973
Clusterin	242	6	52,921
Calmodulin-1	217	4	16,827
Zinc-alpha-2-glycoprotein	179	3	34,421
Trypsin-3	169	3	33,152
Cystatin-S	148	2	16,434
Lysozyme C	138	3	16,894
Alpha-1-acid glycoprotein 1	124	2	23,681
Cathepsin B	118	3	38,579
Mucin-6	117	2	261,946

Cysteine-rich secretory protein 1	97	2	29,244
Midkine	95	1	16,035
Ubiquitin-40S ribosomal protein S27a	94	1	18,229
Glycodelin	92	1	20,886
Antileukoproteinase	88	3	15,052
Metalloproteinase inhibitor 1	82	1	282,963
Galectin-7	77	2	23,707
